# Effects of cardiovascular lifestyle change on lipoprotein subclass profiles defined by nuclear magnetic resonance spectroscopy

**DOI:** 10.1186/1476-511X-8-26

**Published:** 2009-06-29

**Authors:** David J Decewicz, David M Neatrour, Amy Burke, Mary Jane Haberkorn, Heather L Patney, Marina N Vernalis, Darrell L Ellsworth

**Affiliations:** 1 Integrative Cardiac and Metabolic Health Program, Windber Research Institute, Windber, Pennsylvania, USA; 2 Windber Medical Center, Windber, Pennsylvania, USA; 3 Integrative Cardiac Health Program, Walter Reed Army Medical Center, Washington, DC, USA

## Abstract

**Background:**

Low-density lipoprotein (LDL) cholesterol lowering is a primary goal in clinical management of patients with cardiovascular disease, but traditional cholesterol levels may not accurately reflect the true atherogenicity of plasma lipid profiles. The size and concentration of lipoprotein particles, which transport cholesterol and triglycerides, may provide additional information for accurately assessing cardiovascular risk. This study evaluated changes in plasma lipoprotein profiles determined by nuclear magnetic resonance (NMR) spectroscopy in patients participating in a prospective, nonrandomized lifestyle modification program designed to reverse or stabilize progression of coronary artery disease (CAD) to improve our understanding of lipoprotein management in cardiac patients.

**Results:**

The lifestyle intervention was effective in producing significant changes in lipoprotein subclasses that contribute to CAD risk. There was a clear beneficial effect on the total number of LDL particles (-8.3%, p < 0.05 compared to matched controls), small dense LDL particles (-9.5%, p < 0.05), and LDL particle size (+0.8%; p < 0.05). Likewise, participants showed significant improvement in traditional CAD risk factors such as body mass index (-9.9%, p < 0.01 compared to controls), total cholesterol (-5.5%, p < 0.05), physical fitness (+37.2%, p < 0.01), and future risk for CAD (-7.9%, p < 0.01). Men and women responded differently to the program for all clinically-relevant variables, with men deriving greater benefit in terms of lipoprotein atherogenicity. Plasma lipid and lipoprotein responses to the lifestyle change program were not confounded by lipid-lowering medications.

**Conclusion:**

In *at risk *patients motivated to participate, an intensive lifestyle change program can effectively alter traditional CAD risk factors and plasma lipoprotein subclasses and may reduce risk for cardiovascular events. Improvements in lipoprotein subclasses are more evident in men compared to women.

## Background

The causal role of high blood cholesterol in the pathogenesis of coronary artery disease (CAD) is well established. A large body of research indicates that plasma cholesterol levels are important risk factors for CAD, and a number of clinical trials have shown that aggressive cholesterol management in patients with heart disease leads to significant reductions in cardiovascular events [1,2]. At present, the amount of cholesterol carried by lipoprotein particles, in particular low-density lipoprotein (LDL) particles, is an important parameter for estimating CAD risk; thus, clinical strategies for patients with established disease typically focus on lowering LDL as a primary goal of therapy. Traditional cholesterol levels, however, may not accurately reflect the true atherogenicity of plasma lipid profiles because patients with similar cholesterol levels may have differences in the number and size of lipoprotein particles that transport cholesterol and triglycerides and thus may differ in terms of CAD risk.

A more accurate way to assess cardiovascular risk may be to measure the size and concentration of lipoprotein particles [3]. Early studies of lipoprotein subclass distributions using gradient gel electrophoresis found that increased CAD risk was associated with small, dense LDL (sdLDL, pattern B) particles [4-6]. More recent studies using nuclear magnetic resonance (NMR) spectroscopy, which exploits spectral differences between lipoprotein subspecies to directly quantify particle size and concentration, have shown that small LDL particles and a greater number of LDL particles are associated with CAD development and progression in the general population and in patients with coronary disease [7-10]. Because lipoprotein characteristics may be important for assessing cardiovascular risk [11], it is important to understand how lipoproteins respond during cardiovascular treatment and prevention programs to identify interventions that favorably modify atherogenic lipid profiles.

Lifestyle interventions have shown the substantial health benefits of a low fat diet, increased physical activity, and stress management in reducing traditional risk factors for cardiovascular disease [12,13] and in slowing or reversing the progression of coronary atherosclerosis [14,15]. Cardiac patients who follow healthy lifestyles often show notable improvements in standard lipid profiles, highlighted by dramatic reductions in LDL- and total cholesterol [16-19]. Although lipoprotein profiles can be effectively modified by statin or fibrate therapy [20,21], little is known about how lipoprotein subclasses respond to non-pharmacological CAD interventions. The purpose of this study was to evaluate the impact of an intensive risk factor modification program on lipoprotein subclasses defined by NMR spectroscopy and to improve our understanding of lipoprotein management in patients with heart disease. Our objectives were to (1) measure changes in physiological risk factors for CAD throughout a year-long healthy lifestyle intervention, (2) assess response of NMR-measured lipoprotein subclasses and relate changes to improvement in vascular health, and (3) determine possible benefits of the program in terms of cardiovascular risk.

## Methods

The Institutional Review Board at Windber Medical Center approved the research protocol and informed consent documents. All subjects enrolled in the program volunteered to participate and gave written informed consent. Data reporting follows recommendations of the Transparent Reporting of Evaluations with Nonrandomized Designs (TREND) group [22].

### Subjects

The study population consisted of 73 Caucasian participants (34 women and 39 men) who completed a prospective, nonrandomized intervention designed to stabilize or reverse progression of CAD through dietary changes, exercise, stress management, and group support. Entry criteria were (1) a diagnosis of CAD, which included stable angina, angioplasty, evidence of ≥ 50% luminal narrowing on coronary angiogram, acute myocardial infarction, bypass surgery, or stent placement; or (2) two or more CAD risk factors: high blood pressure (BP) – systolic pressure > 140 mm Hg or diastolic pressure > 90 mm Hg, high total cholesterol (> 200 mg/dL), physician diagnosed diabetes, obesity defined as body mass index (BMI) ≥ 30, or family history of heart disease in parents or siblings.

Prospective participants were recruited by advertisements in regional news media and were accepted into the program only with physician approval. Motivation to commit to following the guidelines of the program for the entire year was part of the acceptance criteria and was assessed by in-depth interviews before admission. Because tobacco use is a significant risk factor for CAD, and breaking a nicotine addiction often requires interventions beyond the scope of the lifestyle change program, patients were required to successfully abstain from smoking for at least three months prior to enrollment.

Control participants (n = 73; 34 women and 39 men) receiving only standard care from their primary physicians were matched to lifestyle participants based on gender, age at baseline within a five-year window, and CAD status (diagnosis of CAD or risk factors) using a prospective individual matching strategy similar to that described by Charpentier et al. [23]. The objective of this method is to achieve a balanced distribution of risk factors between intervention and control patients in nonrandomized clinical trials. Control subjects underwent examinations at baseline, 12 weeks, and 52 weeks, but did not participate in the lifestyle change program or receive healthy lifestyle information.

### Intervention

Details on the Dr. Dean Ornish Program for Reversing Heart Disease have been published elsewhere [24,25]. The intervention consists of four components: (1) a very low fat vegetarian diet (< 10% of calories from fat) with emphasis on whole grains, fruits, vegetables, legumes, and soy products; (2) 180 minutes/week within an individually determined heart rate range of moderate aerobic exercise such as walking, rowing, or water aerobics; (3) one hour of stress management each day, which may include a combination of yoga poses, deep breathing, imagery, meditation, and relaxation; and (4) two one-hour group support sessions per week for the first 12 weeks and one group session per week during the remainder of the year. During the first 12 weeks, program staff met with patients two times each week to maximize adherence to the program guidelines and assess any changes in disease status or adverse events. During the remainder of the program, patients came to the center once a week for an hour of stress management and an hour of group support. Patients were primarily self-directed but met with program staff one time each week to review status and progress. From January 2004 to August 2008, approximately 36 patients or controls were enrolled each year in separate cohorts of ~12 individuals per cohort.

All participants in the program were required to submit a personal awareness log each week, which summarized for each day their diet (daily fat, carbohydrate, protein intake calculated as a percentage of calories), exercise (frequency and duration), stress management (frequency and duration), and group support (frequency of meeting attendance). For each component, adherence to the program guidelines was calculated as a percentage of the recommended goals achieved by each patient. Program staff reviewed the compliance forms weekly and provided immediate feedback to patients on their progress and guidance for improving adherence in specific areas as necessary.

Throughout the year-long program, we experienced a drop-out rate of ~32% (n = 46) among participants in the healthy lifestyle program. In addition, 22 participants were excluded from the analysis because no suitable matching control was identified.

### Lipoprotein subclass measurements

Clinical examinations were performed at baseline, 12 weeks, and 52 weeks to assess changes in CAD risk factors and to collect blood for standard lipid and lipoprotein analysis. All examinations were conducted by physicians or trained personnel and followed identical protocols.

Fasting blood samples were obtained at each examination and placed directly on ice. Within one hour of collection, plasma aliquots were isolated from whole blood by centrifugation and stored at -80°C. Lipoprotein subclass profiles were measured on freshly-thawed plasma samples by NMR spectroscopy at LipoScience (Raleigh, NC, USA) following previously published methods [26]. Concentrations of VLDL and LDL (including IDL) subclasses in nmol/L units and HDL subclasses in μmol/L units were obtained from the measured amplitudes of the distinct lipid methyl group NMR signals they emit. The estimated diameters of the nine measured subclasses were as follows: large VLDL (> 60 nm), medium VLDL (35–60 nm), small VLDL (27–35 nm), IDL (23–27 nm), large LDL (21.2–23.0 nm), small LDL (18.0–21.2 nm), large HDL (8.8–13.0 nm), medium HDL (8.2–8.8 nm), and small HDL (7.3–8.2 nm). Total LDL particle concentrations reflect the sum of the IDL, large LDL, and small LDL subclass concentrations, while the sum of large, medium, and small HDL subclass concentrations give total HDL particle concentration. Weighted-average VLDL, LDL, and HDL particle sizes were calculated by summing the diameter of each subclass multiplied by its relative mass percentage as estimated by the amplitude of its methyl NMR signal.

A random sample of patients, selected to assess reproducibility of lipoprotein particle measurements, showed coefficients of variation (CV) between 78 blind duplicate samples that were similar to previously reported values [26]. Coefficients of variation for clinically important lipoprotein parameters were: total LDL particles (6.9%), small LDL particles (13.7%), LDL size (1.5%), large HDL particles (8.1%), HDL size (0.6%), and large VLDL/chylomicrons (18.4%).

### Physiological measures

Participants in the lifestyle change program and prospective controls were interviewed to collect information on age, gender, ethnicity, smoking status, cardiovascular history, and medication use. Height and weight measurements were used to calculate BMI. Blood pressure was recorded using a mercury sphygmomanometer on the arm of seated, relaxed subjects. General endurance was determined by a graded treadmill exercise test that estimated the volume of oxygen each participant could consume (VO_2 _max; ml/kg/min) based on exercise intensity, duration, and body weight (Bruce score) [27]. Assays for standard high-density lipoprotein (HDL) cholesterol, total cholesterol, and triglycerides were conducted using the AEROSET™ multi-task clinical chemistry system (Abbott Laboratories, Abbott Park, IL, USA).

### Cardiovascular risk

Future CAD risk was calculated for each subject as the probability of experiencing a subsequent coronary event in patients with cardiovascular disease or an initial event in disease-free participants within the next four years [28]. The Framingham risk models use separate formulas for men and women. Individuals were excluded from the risk calculations (n = 8) if their CAD status changed due to a cardiac event during the year or if they had missing data in one or more fields needed to calculate CAD risk.

### Statistical analysis

Statistical analyses were conducted using SPSS version 15.0; p values < 0.05 were considered statistically significant. Prior to analysis, Lilliefors test was used to determine normality of the outcome data, and natural log-transformations were used for variables with non-normal distributions. Potential differences in baseline measures among the lifestyle program and control group cohorts were examined by analysis of variance (ANOVA). As no significant cohort-to-cohort variability at baseline was detected, all intervention and control cohorts were respectively combined in subsequent analyses.

Baseline characteristics between lifestyle participants and control subjects were compared by an independent samples Student-t test, or by a nonparametric Mann-Whitney U test if the data remained non-normally distributed after natural log transformation. Statistical comparisons of changes in CAD risk factors at 12 weeks and 52 weeks between the intervention and control groups were done using repeated-measures ANOVA with group (intervention versus control) as the between-subjects factor. Independent samples t-tests (two-tailed) then identified differences in CAD risk factor response from baseline to week 52 between the intervention and control groups. For each outcome variable, differences in response between men and women were assessed by two-factor repeated measures ANOVA (baseline, 12 weeks, and 52 weeks) with group and gender within groups as the between-subjects factors, using a Bonferroni adjustment for multiple comparisons by time point, group, and gender within group. As above, independent samples t-tests (two-tailed) compared baseline to week 52 changes between groups, by gender. To examine the potential confounding effects of lipid-lowering medications on response to the program, a sub-group analysis was conducted that included only participants who continued taking the same brand and dosage of lipid-lowering medications as well as those not taking any lipid-lowering drugs.

## Results

Baseline characteristics of participants in the lifestyle change program and matched controls are presented in Table 1. Intervention subjects differed from controls for many lipoprotein subclass measurements and several physiological variables despite the prospective matching strategy, which matched controls to participants based on gender, age at entry, and CAD status. For clinically relevant measures that may contribute to CAD risk, participants in the program usually had a more atherogenic risk factor profile.

**Table 1:** Lipoprotein subclass measurements and physiological variables at baseline by case/control status

Variable	Controls (n = 73)	Participants (n = 73)	p value^a^
**Lipoprotein subclass**^b^			
VLDL and chylomicron particle concentrations (nmol/L)			
Total VLDL and chylomicron particles	82.0 ± 45.3	87.5 ± 39.6	0.15
Large VLDL and chylomicron particles	4.6 ± 6.3	8.2 ± 6.8	< 0.01^c^
Medium VLDL particles	38.1 ± 28.5	39.7 ± 25.7	0.50^c^
Small VLDL particles	39.3 ± 19.9	39.7 ± 17.2	0.90
			
LDL particle concentrations (nmol/L)			
Total LDL particles	1253 ± 367	1437 ± 477	0.01
Large LDL particles	337 ± 211	251 ± 218	< 0.01^c^
Intermediate LDL particles	45.8 ± 38.5	72.6 ± 50.2	< 0.01^c^
Small LDL particles	870 ± 434	1113 ± 478	< 0.01
			
HDL particle concentrations (μmol/L)			
Total HDL particles	34.7 ± 6.7	32.1 ± 5.6	0.01
Large HDL particles	6.4 ± 3.5	4.6 ± 3.4	< 0.01
Medium HDL particles	5.2 ± 4.9	5.9 ± 4.8	0.25^c^
Small HDL particles	23.1 ± 7.0	21.7 ± 5.8	0.17
			
Mean particle sizes (nm)			
VLDL size	50.6 ± 9.0	55.6 ± 10.4	< 0.01
LDL size	20.7 ± 0.9	20.2 ± 0.8	< 0.01
HDL size	8.9 ± 0.4	8.6 ± 0.3	< 0.01
			
**Physiological variables**^d^			
Age	60.0 ± 7.8	60.3 ± 8.0	0.79
Body mass index (kg/m^2^)	28.2 ± 3.7	34.0 ± 7.0	< 0.01
Systolic blood pressure (mm Hg)	133 ± 17	136 ± 17	0.12^c^
Diastolic blood pressure (mm Hg)	78.9 ± 9.2	80.8 ± 9.8	0.50^c^
HDL-cholesterol (mg/dl)	50.5 ± 13.7	43.7 ± 13.1	< 0.01^c^
LDL-cholesterol (mg/dl)	111 ± 33	111 ± 39	0.64^c^
Total cholesterol (mg/dl)	191 ± 42	193 ± 47	0.82
Triglycerides (mg/dl)	148 ± 100	189 ± 99	< 0.01
Physical fitness (Bruce score)	10.4 ± 2.8	6.6 ± 2.2	< 0.01
Framingham risk (× 100)	7.4 ± 6.8	10.7 ± 8.5	0.02^c^

Values are presented as mean ± SD. a: tested by independent samples Student-t test, b: there was no missing data, c: tested by a nonparametric Mann-Whitney U test because data was not normally distributed after LN transformation, d: there was < 3.2% missing data.

Lipoprotein subclass measurements did not differ significantly between participants who completed the lifestyle intervention (graduates) and those who dropped out. For traditional CAD risk factors, dropouts tended to be younger (55.0 ± 11.2 versus 60.3 ± 8.0; p < 0.01) and have lower systolic BP (130.1 ± 18.8 versus 136.5 ± 17.0; p < 0.05) than graduates. Participants excluded from the analysis due to non-matching were older (64.6 ± 10.8 compared to 60.3 ± 8.0; p < 0.05) and had larger HDL particles (8.8 ± 0.4 versus 8.6 ± 0.3; p < 0.05) and higher HDL-cholesterol (49.6 ± 11.1 versus 43.7 ± 13.1; p < 0.01) than participants included in the study.

### Effects of lifestyle changes on lipoproteins

**Figure 1:**
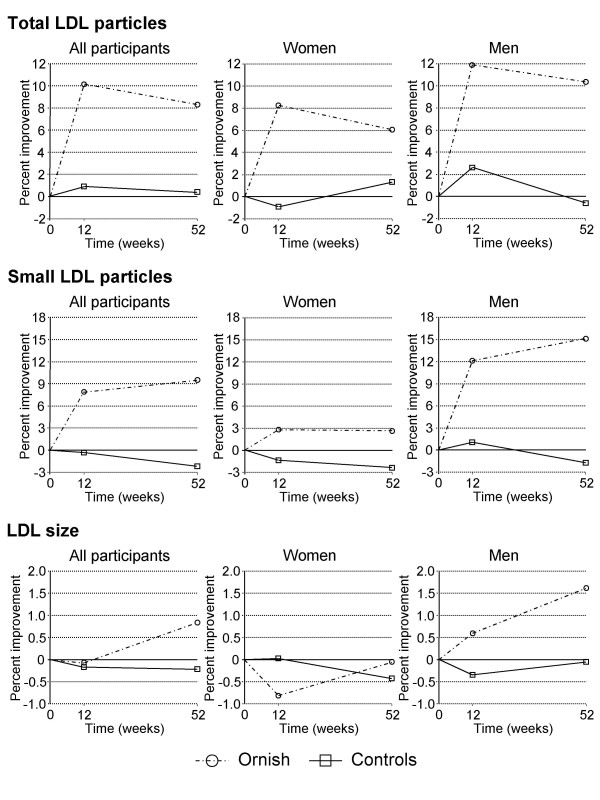
**Changes in LDL particle concentrations during the lifestyle change program**. All participants were included in the initial analyses (left panels), then stratified by gender (right panels). Improvement: decrease in total and small LDL particles; increase in LDL size.

The lifestyle intervention led to significant changes in NMR-defined lipoprotein subclasses, in particular, clinically important LDL variables that contribute to CAD risk (Figure 1). There was a clear beneficial effect on the total number of LDL particles, which decreased 8.3% overall in participants (p < 0.05 compared to controls). Likewise, sdLDL particles, which have been associated with CAD risk in several studies, decreased 9.5% during the program (p < 0.05 versus controls). Lifestyle changes also were effective in increasing both HDL (+1.0%; p < 0.01) and LDL (+0.8%; p < 0.05) particle size, while decreasing very-low-density lipoprotein (VLDL) size (-9.7%; p < 0.01) and large VLDL and chylomicron concentrations (-29.4%; p < 0.05) (Table 2).

**Table 2:** Changes in lipoprotein subclass measurements and physiological variables by case/control status

	Controls (n = 73)				Participants (n = 73)				
Variable	Baseline	Week 12	Week 52	% change	Baseline	Week 12	Week 52	% change	Between group p value^a^
**Lipoprotein subclass^b^**									
VLDL and chylomicron particles (nmol/L)									
Tot VLDL/chylo	82.0 ± 45.3	80.2 ± 42.6	81.9 ± 47.2	-0.2	87.5 ± 39.6	96.2 ± 43.3	88.5 ± 45.3	+1.2	0.84
Lg VLDL/chylo	4.6 ± 6.3	5.2 ± 6.2	4.5 ± 5.4	-1.2	8.2 ± 6.8	4.7 ± 5.3^c^	5.8 ± 7.0^d^	-29.4	0.03
Medium VLDL	38.1 ± 28.5	37.4 ± 24.3	37.8 ± 29.5	-0.9	39.7 ± 25.7	49.8 ± 31.8^c^	42.0 ± 28.6	+6.0	0.50
Small VLDL	39.3 ± 19.9	37.6 ± 22.1	39.6 ± 19.9	+0.6	39.7 ± 17.2	41.7 ± 18.7	40.7 ± 21.7	+2.6	0.80
									
LDL particles (nmol/L)									
Total LDL	1253 ± 367	1242 ± 422	1249 ± 414	-0.4	1437 ± 477	1291 ± 445^c^	1317 ± 452^d^	-8.3	0.04
Large LDL	337 ± 211	315 ± 191	317 ± 208	-5.8	251 ± 218	215 ± 152	263 ± 174	+4.5	0.24
Intermed LDL	45.8 ± 38.5	55.6 ± 56.2	43.1 ± 44.6	-5.8	72.6 ± 50.1	50.6 ± 43.4^c^	47.3 ± 46.3^c^	-34.9	< 0.01
Small LDL	870 ± 434	872 ± 445	888 ± 457	+2.1	1113 ± 478	1025 ± 432	1007 ± 470^d^	-9.5	0.04
									
HDL particles (μmol/L)									
Total HDL	34.7 ± 6.7	35.2 ± 6.1	35.2 ± 6.4	+1.7	32.1 ± 5.6	29.0 ± 5.0^c^	32.3 ± 5.9	+0.6	0.64
Large HDL	6.4 ± 3.5	6.7 ± 3.3	6.5 ± 3.5	+1.4	4.6 ± 3.4	4.3 ± 2.2	5.2 ± 2.9	+12.7	0.19
Medium HDL	4.9 ± 4.6	5.2 ± 5.1	4.5 ± 4.5	-8.6	5.6 ± 4.7	4.2 ± 4.1^c^	5.2 ± 5.3	-7.8	0.70
Small HDL	23.1 ± 7.0	23.0 ± 7.0	24.2 ± 6.7	+4.6	21.7 ± 5.8	20.6 ± 4.7	22.1 ± 6.9	+2.2	0.49
									
Mean particle sizes (nm)									
VLDL size	50.6 ± 9.0	51.8 ± 8.2	50.9 ± 7.5	+0.6	55.6 ± 10.4	48.2 ± 8.6^c^	50.2 ± 9.5^c^	-9.7	< 0.01
LDL size	20.7 ± 0.9	20.7 ± 0.8	20.7 ± 0.8	-0.2	20.2 ± 0.8	20.2 ± 0.6	20.4 ± 0.7	+0.8	0.03
HDL size	8.8 ± 0.4	8.9 ± 0.4	8.8 ± 0.4	-0.4	8.6 ± 0.3	8.7 ± 0.3	8.7 ± 0.4^d^	+1.0	< 0.01
									
**Physiological variables**^e^									
BMI	28.2 ± 3.7	28.0 ± 3.9	28.3 ± 3.8	+0.4	34.0 ± 7.0	31.4 ± 6.3^c^	30.7 ± 6.6^c^	-9.9	< 0.01
Systolic BP	133 ± 17	128 ± 15^d^	126 ± 13^d^	-5.4	136 ± 17	122 ± 14^c^	127 ± 16^c^	-6.6	0.55
Diastolic BP	78.9 ± 9.2	78.1 ± 8.0	77.3 ± 8.4	-2.0	80.8 ± 9.8	72.6 ± 8.3^c^	76.0 ± 9.4^d^	-6.0	0.08
HDL	50.5 ± 13.7	51.9 ± 12.7	48.1 ± 13.5^d^	-4.9	43.7 ± 13.1	37.5 ± 9.0^c^	42.8 ± 10.3	-2.1	0.26
LDL	111 ± 33	110 ± 35	112 ± 36	+1.0	111 ± 39	96 ± 34^c^	107 ± 34	-3.9	0.23
Tot cholesterol	191 ± 42	192 ± 45	191 ± 46	+0.2	193 ± 47	167 ± 44^c^	183 ± 43^d^	-5.5	0.03
Triglycerides	148 ± 100	158 ± 140	152 ± 89	+2.8	189 ± 99	167 ± 73	168 ± 97	-11.3	0.05
Bruce	10.4 ± 2.8	10.6 ± 2.7	10.3 ± 2.8	-1.4	6.6 ± 2.2	8.5 ± 2.3^c^	9.0 ± 2.7^c^	+37.2	< 0.01
Fram risk	7.2 ± 6.7	6.8 ± 6.5	7.2 ± 7.2	+0.6	11.0 ± 8.6	10.2 ± 8.5^d^	10.1 ± 7.8^d^	-7.9	< 0.01

Values are presented as mean ± SD; differences between table values and calculated values are due to rounding; % change = week 0–52. a: p values resulting from independent samples t-tests (two-tailed) of Baseline to Week 52 changes in Ornish participants compared to the control group, b: there was no missing data, c: values at Week 12 and Week 52 were significantly different from Baseline at p < 0.001 based on repeated-measures ANOVA with time point as the within-subjects factor and cohort type as the between-subjects factor, d: values at Week 12 and Week 52 were significantly different from Baseline at p < 0.05 based on repeated-measures ANOVA with time point as the within-subjects factor and cohort type as the between-subjects factor, e: there was < 4.2% missing data.

### Lifestyle changes and traditional risk factor response

Participation in the lifestyle change program had significant beneficial effects on traditional (physiological) CAD risk factors (Table 2). Patients achieved on average a 9.9% reduction in BMI by the end of the year-long program (p < 0.01 compared to controls), a 5.5% reduction in total cholesterol (p < 0.05), and a 37.2% increase in their physical fitness score (p < 0.01). No significant differences in response at 52 weeks between cases and controls were observed for HDL- and LDL-cholesterol. Although program participants showed a significant decrease (-14%; p < 0.001) in HDL-cholesterol from baseline to 12 weeks, by the end of the year HDL recovered to near baseline levels. Benefits of the program were also evident in changes in future risk for CAD – average decrease in Framingham risk among participants was 7.9% compared to a 0.6% increase in controls (p < 0.01).

### Gender differences in response

For variables that showed a significant difference in outcome between participants and controls, we used repeated measures ANOVA by group and gender nested within group to investigate gender specific responses. Gender was not a significant factor for changes in lipoprotein subclasses from baseline to 52 weeks among controls; however, men and women participating in the program responded differently for all clinically-relevant variables (Table 3). Among female participants, changes in clinically-important lipoprotein measures did not differ significantly from women in the control group, and were not different from baseline after 52 weeks. In contrast, men following the healthy lifestyle appeared to derive greater benefit than women in terms of lipoprotein atherogenicity (Figure 1). Specifically, total LDL particle concentrations (-10.4%; p < 0.05 compared to control men), sdLDL (-15.1%; p < 0.01 versus men in the control group), LDL particle size (+1.6%; p < 0.01), and large VLDL (-55.9%; p < 0.001) all improved significantly among men who participated in the program. Similar patterns of change also were apparent for large HDL particles (+34.9%; p < 0.05) and HDL size (+1.6%; p < 0.05).

**Table 3:** Changes in selected lipoprotein subclass measurements and standard plasma lipid profiles by gender

		Controls (n = 73)				Participants (n = 73)				
Variable	Gender	Baseline	Week 12	Week 52	% change	Baseline	Week 12	Week 52	% change	Between group p value^a^

**VLDL particles**										
Large VLDL/chylomicrons	F	5.7 ± 7.7	6.1 ± 7.4	5.0 ± 6.0	-12.4	7.9 ± 7.0	5.9 ± 6.2	8.1 ± 8.5	+3.0	0.62
	M	3.6 ± 4.5	4.4 ± 5.0	4.1 ± 4.9	+14.5	8.4 ± 6.8	3.6 ± 4.1^b^	3.7 ± 4.7^b^	-55.9	< 0.01
										
**LDL particles**										
Total LDL particles	F	1364 ± 388	1376 ± 500	1347 ± 475	-1.3	1478 ± 497	1356 ± 460	1388 ± 516	-6.1	0.42
	M	1156 ± 323	1126 ± 302	1163 ± 335	+0.6	1401 ± 463	1234 ± 430^c^	1256 ± 385^c^	-10.4	0.03
										
Small LDL particles	F	902 ± 491	914 ± 548	924 ± 534	+2.4	1076 ± 565	1046 ± 485	1048 ± 568	-2.6	0.63
	M	843 ± 382	834 ± 334	857 ± 381	+1.7	1145 ± 393	1006 ± 385^c^	972 ± 368^c^	-15.1	< 0.01
										
LDL size	F	20.9 ± 0.9	20.9 ± 0.9	20.8 ± 0.9	-0.4	20.5 ± 0.9	20.4 ± 0.7	20.5 ± 0.8	0.0	0.62
	M	20.6 ± 0.8	20.5 ± 0.7	20.5 ± 0.7	0.0	20.0 ± 0.6	20.1 ± 0.6	20.3 ± 0.6^c^	+1.6	< 0.01
										
**HDL particles**										
Large HDL particles	F	7.4 ± 3.7	7.8 ± 3.5	7.4 ± 3.8	-0.3	5.8 ± 4.1	5.1 ± 2.6^c^	5.7 ± 3.1	-2.6	0.83
	M	5.4 ± 3.0	5.7 ± 2.8	5.6 ± 2.9	+3.3	3.5 ± 2.1	3.7 ± 1.4	4.7 ± 2.7^c^	+34.9	0.03
										
HDL size	F	9.0 ± 0.4	9.0 ± 0.4	8.9 ± 0.4	-0.7	8.8 ± 0.3	8.7 ± 0.2	8.8 ± 0.3	+0.4	0.07
	M	8.7 ± 0.4	8.8 ± 0.4	8.7 ± 0.4	0.0	8.5 ± 0.2	8.6 ± 0.2^c^	8.6 ± 0.4^c^	+1.6	0.03
										
**Lipid profiles**										
HDL-cholesterol	F	55.9 ± 12.6	57.2 ± 11.8	52.9 ± 13.0	-5.3	49.2 ± 15.5	40.7 ± 10.5^b^	45.5 ± 9.3^c^	-7.4	0.77
	M	45.9 ± 13.0	47.3 ± 11.7	43.8 ± 12.6	-4.5	38.9 ± 8.1	34.8 ± 6.6^c^	40.4 ± 10.6	+3.8	0.03
										
Tot cholesterol	F	211 ± 35	213 ± 44	208 ± 43	-1.4	206 ± 48	181 ± 42^b^	197 ± 43	-4.5	0.42
	M	173 ± 40	173 ± 38	177 ± 44	+2.0	182 ± 45	156 ± 43^b^	171 ± 41	-6.4	0.02
										
Triglycerides	F	164 ± 105	181 ± 193	157 ± 85	-3.9	192 ± 100	188 ± 83	199 ± 117	+3.9	0.54
	M	134 ± 95	139 ± 62	148 ± 94	+9.8	187 ± 100	149 ± 59^c^	140 ± 65^c^	-24.9	< 0.01

Values are presented as mean ± SD; differences between table values and calculated values are due to rounding; % change = week 0–52. There were 34 women and 39 men in each group, there was no missing data. a: p values resulting from independent samples t-tests (two-tailed) of Baseline to Week 52 changes for female Ornish participants compared to female controls and male Ornish participants compared to male controls, b: values at Week 12 and Week 52 were significantly different from Baseline at p < 0.001 based on repeated measures ANOVA (time point was the within-subjects factor, cohort type and gender within cohorts were the between-subjects factors) using a Bonferroni adjustment for multiple comparisons by time point, group, and gender within group, c: values at Week 12 and Week 52 were significantly different from Baseline at p < 0.05 based on repeated measures ANOVA (time point was the within-subjects factor, cohort type and gender within cohorts were the between-subjects factors) using a Bonferroni adjustment for multiple comparisons by time point, group, and gender within group.

For physiological variables, both men and women showed a significant decrease in BMI and increase in physical fitness scores (p < 0.001) compared to controls after one year. Similar to lipoproteins, however, HDL-cholesterol (+3.8%; p < 0.05), triglycerides (-24.9%; p < 0.001), and total cholesterol (-6.4%; p < 0.05) improved significantly in male participants compared to male controls, but no significant changes compared to controls were seen in women.

### Effects of lipid-lowering medications

Lipid-lowering therapy did not have significant effects on lipid and lipoprotein responses to the lifestyle change program. There were 56 participants (77%) and 47 controls (64%) who were not taking lipid-lowering drugs or whose medication levels did not change during the study. Results of the sub-group analysis examining lipid and lipoprotein responses in these patients were largely similar to analyses encompassing all participants (Table 4). When controlling for changes in medications, some variables were no longer significantly different from baseline at 52 weeks, but still showed significant improvement compared to controls with no changes in lipid-lowering medications.

**Table 4:** Effects of lipid-lowering medication changes on selected variables after 52 weeks

		All participants			No medication changes		
			
		% change (wk 0–52)			% change (wk 0–52)		
					
Variable	Gender	Controls (n = 73)	Ornish (n = 73)	Between group p value^a^	Controls (n = 47)	Ornish (n = 56)	Between group p value^a^
**VLDL particles**							
Large VLDL and chylomicrons	F	-12.4	+3.0	0.62	+4.9	+4.6	0.95
	M	+14.5	-55.9^b^	< 0.01	+26.9	-54.9^b^	< 0.01
							
**LDL particles**							
Total LDL particles (nmol/L)	F	-1.3	-6.1	0.42	-1.2	-4.2	0.63
	M	+0.6	-10.4^c^	0.03	+8.1	-3.8	0.02
							
Small LDL particles (nmol/L)	F	+2.4	-2.6	0.63	+1.3	-0.9	0.86
	M	+1.7	-15.1^c^	< 0.01	+13.2	-8.6	< 0.01
							
LDL size (nm)	F	-0.4	0.0	0.62	-0.4	0.0	0.70
	M	0.0	+1.6^c^	< 0.01	-0.6	+1.5^c^	< 0.01
							
**HDL particles**							
Large HDL particles (μmol/L)	F	-0.3	-2.6	0.83	+0.3	-4.2	0.71
	M	+3.3	+34.9^c^	0.03	-1.2	+31.6	0.04
							
HDL size (nm)	F	-0.7	+0.4	0.07	-0.8	+0.4	0.15
	M	0.0	+1.6^c^	0.03	-0.5	+0.8	0.08
							
**Lipid profiles**							
HDL-cholesterol	F	-5.3	-7.4^c^	0.77	-6.5	-9.0^c^	0.83
	M	-4.5	+3.8	0.03	-4.3	+3.3	0.08
							
Total cholesterol	F	-1.4	-4.5	0.42	-0.2	-3.2	0.47
	M	+2.0	-6.4	0.02	+5.7	-2.9	< 0.01
							
Triglycerides	F	-3.9	+3.9	0.54	+5.1	+5.4	0.91
	M	+9.8	-24.9^c^	< 0.01	+10.1	-23.5^c^	< 0.01

There were 34 female and 39 male participants in the control and intervention groups, 19 female and 28 male controls with no medication changes, 30 female and 26 male participants with no medication changes. a: p values resulting from independent samples t-tests (two-tailed) of Baseline to Week 52 changes for female Ornish participants compared to female controls and male Ornish participants compared to male controls, b: percent changes from Baseline to Week 52 were significant at p < 0.001 based on repeated measures ANOVA (time point was the within-subjects factor, cohort type and gender within cohorts were the between-subjects factors) using a Bonferroni adjustment for multiple comparisons by time point, group, and gender within group, c: percent changes from Baseline to Week 52 were significant at p < 0.05 based on repeated measures ANOVA (time point was the within-subjects factor, cohort type and gender within cohorts were the between-subjects factors) using a Bonferroni adjustment for multiple comparisons by time point, group, and gender within group.

## Discussion

Participants who completed a comprehensive year-long lifestyle change program designed to reverse or stabilize progression of CAD showed significant improvement in traditional cardiovascular risk factors and a significant decrease in future cardiovascular risk. The lifestyle intervention also was effective in improving lipoprotein profiles that contribute to CAD risk. When analyses were stratified by gender, men appeared to derive greater vascular health benefit than women due to significant changes in important lipoprotein subclasses, including decreases in LDL particle number, sdLDL particles, and large VLDL, and increases in HDL and LDL particle size. Women showed no significant improvement for any of the measured lipoprotein parameters. Changes in clinically-relevant LDL particle attributes were comparable to other behavioral and pharmacologic interventions for lipoprotein management and were not significantly influenced by changes in lipid-lowering medications.

Lifestyle modification programs are known to improve cardiovascular risk profiles through significant reductions in LDL- and total cholesterol, but the effects of lifestyle interventions on lipoprotein profiles and vascular health are not well known. To date, only limited research has examined the impact of lifestyle behaviors, such as exercise and diet, on lipoprotein subclass distributions. Meta-analyses suggest that aerobic exercise has modest effects on blood cholesterol levels [29,30], but can lead to important beneficial changes in lipoprotein subfractions, including a reduction in the number of LDL particles and an increase in both HDL and LDL particle size [31,32]. Similarly, low-fat – high-carbohydrate diets targeting weight loss have shown favorable effects on lipoprotein particle size and distribution [33,34]. When dietary interventions are used in combination with an exercise regimen, patients show significant increases in particle size and a shift toward larger LDL particles [35].

To make lifestyle interventions more comprehensive, programs have been developed to focus on numerous aspects of lifestyle including weight management, physical activity, nutrition, smoking cessation, and stress management. These multidisciplinary programs have been shown to improve modifiable cardiovascular risk factors [16-19], but effects on lipoprotein profiles are not well known. Compared to controls receiving usual care, we observed clear benefit to participants for many of the lipoprotein parameters measured by NMR spectroscopy. Although lipoproteins are not used in the Framingham formulas for determining CAD risk, successful modulation of lipoproteins through lifestyle changes may have positive effects on vascular health not readily apparent from traditional risk factor profiles.

Lipoproteins play an important role in the development and progression of atherosclerotic disease because lipoprotein particles transport cholesterol and triglycerides throughout the body. In particular, low density lipoproteins are the primary carriers of cholesterol in plasma, but are not readily cleared from the circulation. Longer residence time in the vasculature leads to increased uptake by the arterial walls and greater susceptibility to oxidization. LDL particles modified by oxidation accelerate disease progression by promoting foam cell formation, inflammation, and endothelial dysfunction [36].

In patients with an atherogenic lipoprotein profile characterized by a high number of LDL particles, an abundance of sdLDL particles, and small LDL size, the vascular intima is constantly exposed to high concentrations of lipoproteins, which interact with the arterial walls and initiate the cascade of events leading to atherosclerosis [37]. Numerous studies have shown that the total number of LDL particles is a strong independent predictor of CAD risk [7-9]. Likewise, an abundance of sdLDL particles (and thus lower average LDL particle size) is associated with a ~3-fold increase in risk of heart disease in univariate analyses (reviewed in [36]). sdLDL particles are thought to be particularly atherogenic because smaller particles: (1) readily penetrate arterial tissue [38], (2) are susceptible to oxidation [39], and (3) easily bind to arterial proteoglycans [40]. For patients with atherosclerotic disease, favorable changes in lipoprotein subclass distributions may decrease vascular reactivity and endothelial dysfunction, thereby arresting progression of atherosclerosis. Significant reductions in the total number of LDL and sdLDL particles would minimize the number of atherogenic particles available to interact with the arterial walls, resulting in a reduction in foam cell formation and less vascular inflammation.

Despite evidence that behavioral changes can lead to significant improvements in atherogenic lipid profiles, certain at-risk patients may not show comparable benefit [41]. Women in particular may not respond favorably to low-fat diet and exercise regimens in terms of plasma lipid parameters [42] and may be less compliant than men in meeting lifestyle program goals [43]. In other studies, men achieved greater reductions in total cholesterol concentrations than women in response to a fat-restricted diet [44]. Here, men enrolled in the Ornish Program showed a favorable decrease in LDL particle number, sdLDL concentrations, and large VLDL, as well as an increase in HDL and LDL size; however, comparable changes were not observed in women. Of all lipoprotein and physiological variables, women showed significant improvements relative to controls only for BMI and physical fitness. Our data thus suggest that men may derive greater vascular health benefits from a comprehensive lifestyle modification program. Interestingly, of the four program components – diet, exercise, stress management, and group support – only dietary compliance (%) at 52 weeks differed significantly (p < 0.05) between men (96.7 ± 2.6) and women (94.5 ± 4.9). Thus gender specific differences in lipoprotein response to the program may be attributable to endogenous hormones that affect lipoprotein storage, transport, and metabolism [45].

Successful stabilization and regression of established atherosclerotic plaques may be achievable clinical goals, but many interventions fail to induce regression of advanced lesions containing necrotic, fibrotic, and calcified tissues because patients cannot achieve the drastic changes in plasma lipoproteins necessary to stabilize the plaque environment [46]. Pharmacologic agents are commonly used to lower lipoprotein concentrations, but patients often exhibit tremendous variability in response to different drugs [47]. Our data suggest that changes in several LDL particle attributes among participants in the lifestyle change program were comparable to, or superior to, responses reported from other behavioral and pharmacologic interventions [9,20,21,31,32,34,35,48-56] (Table 5). For example, men participating in the program showed significant reductions in LDL particle number (-10.4%) and sdLDL particle concentrations (-15.1%), which were similar to or exceeded exercise training and fibrate or thiazolidinedione (TZD) therapy. Fibrates (gemfibrozil, fenofibrate) are used to modulate plasma lipids in patients with high cholesterol [57], while TZDs (pioglitazone, rosiglitazone) are insulin-sensitizing drugs commonly used to treat non-insulin-dependent diabetes [58]. Likewise, the increase in LDL size (+1.6%) in male participants was greater percentage-wise than that attributable to behavioral interventions or statins (atorvastatin, pravastatin), which are widely prescribed to lower blood cholesterol and prevent cardiovascular disease [59].

**Table 5:** Response of LDL particle number, LDL size, and small LDL particles to pharmacologic and lifestyle interventions

LDL subclass^a^	Trial/Center	Intervention	Daily dosage(mg)	Mean follow-up (weeks)	Baseline value ± SD	Mean change (%)	Reference
**Total LDL particles**	GLAI^b^	rosiglitazone	4/8	24	1368 ± 372	+8.2	[48]
**(nmol/L)**	GLAI^b^	pioglitazone	30/45	24	1394 ± 361	-3.5	[48]
	STRRIDE^c^	exercise	---	~35	1456 ± 86^d^	-4.5	[31]
	VA-HIT^e^	gemfibrozil	1200	~30	1352 ± 316	-4.6	[21]
	JUSMH^f^	bezafibrate	400	4	1722 ± 629	-4.6	[49]
	Ornish Program^g^	lifestyle (F)	---	52	1478 ± 497	-6.1	
	UMCP^h^	exercise	---	24	1436 ± 42^d^	-7.0	[32]
	Ornish Program^g^	lifestyle (M)	---	52	1401 ± 463	-10.4	
	MC^i^	pioglitazone	30	19	1420 ± 74^d^	-10.6	[35]
	JUSMH^j^	fenofibrate	200	8	1567 ± 606	-10.9	[50]
	COMPLEMENT^k^	pioglitazone	30/45	17	1527 ± 473	-12.4	[51]
	VCU^l^	niacin IR^m^	3000	12	2561 ± 81^d^	-14.1	[52]
	DU^n^	low-fat diet	---	24	1759	-14.7	[34]
	TJU^o^	niacin ER^p^	1000	12	1993	-15.0	[53]
	MC^i^	diet/exercise	---	19	1216 ± 55^d^	-18.8	[35]
	PRINCE^q^	pravastatin	40	12	1540^r^	-19.0^r^	[54]
	TJU^o^	niacin ER^p^	2000	12	2048	-23.0	[53]
	PLAC-1^s^	pravastatin	20–40	~26	1908 ± 304	-24.0	[20]
	PLAC-1^t^	pravastatin	40	~26	1918 ± 292	-25.5	[9]
	VCU^l^	atorvastatin	10	12	2562 ± 77^d^	-31.4	[52]
	CARDS^u^	atorvastatin	10	26	1572^r^	-31.6^r^	[55]
							
**LDL size**	CARDS^u^	atorvastatin	10	26	20.6^r^	-0.7^r^	[55]
**(nm)**	Ornish Program^g^	lifestyle (F)	---	52	20.5 ± 0.9	0.0	
	PLAC-1^s^	pravastatin	20–40	~26	20.7 ± 0.5	+0.3	[20]
	PLAC-1^t^	pravastatin	40	~26	20.7 ± 0.4	+0.5	[9]
	PRINCE^q^	pravastatin	40	12	20.8^r^	+0.5^r^	[54]
	STRRIDE^c^	exercise	---	~35	20.8 ± 0.2^d^	+1.0	[31]
	UMCP^h^	exercise	---	24	21 ± 0.1^d^	+1.0	[32]
	COMPLEMENT^k^	pioglitazone	30/45	17	20.3 ± 0.7	+1.1	[51]
	DU^n^	low-fat diet	---	24	20.9	+1.4	[34]
	VCU^l^	atorvastatin	10	12	19.8 ± 0.1^d^	+1.5	[52]
	MC^i^	diet/exercise	---	19	20.6 ± 0.2^d^	+1.5	[35]
	Ornish Program^g^	lifestyle (M)	---	52	20.0 ± 0.6	+1.6	
	GLAI^b^	rosiglitazone	4/8	24	20.1 ± 0.8	+1.6	[48]
	TJU^o^	niacin ER^p^	1000	12	20.0	+2.0	[53]
	MC^i^	pioglitazone	30	19	20.3 ± 0.2^d^	+2.0	[35]
	TJU^o^	niacin ER^p^	2000	12	21.0	+2.0	[53]
	GLAI^b^	pioglitazone	30/45	24	20.0 ± 0.8	+2.3	[48]
	VCU^l^	niacin IR^m^	3000	12	19.9 ± 0.1^d^	+2.5	[52]
	VA-HIT^e^	gemfibrozil	1200	~30	20.4 ± 0.8	+2.5	[21]
	JUSMH^j^	fenofibrate	200	8	19.7 ± 0.8	+3.8	[50]
	JUSMH^f^	bezafibrate	400	4	19.9 ± 1.0	+3.9	[49]
	NEMC/UPSM^v^	torcetrapib	120	4	20.4 ± 0.9	+4.9	[56]
	NEMC/UPSM^v^	torcetrapib	120/240	8	20.4 ± 0.8	+7.4	[56]
							
**Small LDL**	CARDS^u^	atorvastatin	10	26	599^r^	+1.8^r^	[55]
**particles (nmol/L)**	Ornish Program^g^	lifestyle (F)	---	52	1076 ± 565	-2.6	
	GLAI^b^	rosiglitazone	4/8	24	1142 ± 429	-3.4	[48]
	UMCP^h^	exercise	---	24	966 ± 55^d^	-13.4	[32]
	Ornish Program^g^	lifestyle (M)	---	52	1145 ± 393	-15.1	
	COMPLEMENT^k^	pioglitazone	30/45	17	1188 ± 513	-19.3	[51]
	VA-HIT^e^	gemfibrozil	1200	~30	967 ± 406	-19.6	[21]
	MC^i^	pioglitazone	30	19	1119 ± 95^d^	-20.2	[35]
	MC^i^	diet/exercise	---	19	888 ± 75^d^	-27.3	[35]

a: determined by NMR spectroscopy, b: 333 (pioglitazone) or 325 (rosiglitazone) type 2 diabetic men and women with dyslipidemia, following 4-week washout period intervention was divided into two 12-week intervals where dosage increased in second interval, subjects instructed to follow American Heart Association Step One diet throughout the intervention, c: Studies of Targeted Risk Reduction Interventions through Defined Exercise – 22 sedentary, overweight men and women with mild to moderate dyslipidemia (high-amount – high-intensity exercise group), d: values are mean ± SE, e: Veterans Affairs High-Density Lipoprotein Intervention Trial – 515 men with known coronary heart disease, f: Jikei University School of Medicine Hospital – hypertriglyceridemic men (22) and women (2), g: 39 men (M) or 34 women (F) with overt CAD or risk factors who participated in the lifestyle change program, h: University of Maryland, College Park – sedentary healthy men (42) and women (58), subjects followed the American Heart Association Dietary Guidelines for the General Population prior to and throughout exercise training, i: Mayo Clinic – non-diabetic insulin-resistant men (18) and women (19), patients consumed an isocaloric diet for one week prior to baseline and follow-up, j: Jikei University School of Medicine Hospital – 20 hypertriglyceridemic men, k: 295 (baseline) type 2 diabetic men and women with dyslipidemia who received stable doses of rosiglitazone and statins for > 90 days, at enrollment rosiglitazone was discontinued and 30 mg once daily pioglitazone was initiated – at the end of the study, 52% were receiving 30 mg/day, while 48% were receiving 45 mg/day, l: Virginia Commonwealth University – 53 (atorvastatin) or 48 (niacin) men and women with atherogenic dyslipidemia, treatment followed a 6-week lead-in period on a National Cholesterol Education Program Step One diet, m: immediate release (IR), n: Duke University – 60 obese, hyperlipidemic men and women who consumed a low-calorie (1590 kcal/day), low-fat (54 g fat/day) diet for 24 weeks, o: Thomas Jefferson University – 21 (1000 mg) or 20 (2000 mg) men and women with primary hypercholesterolemia, treatment followed an 8-week lead-in phase on an American Heart Association Step One diet, p: extended release (ER), q: Pravastatin Inflammation/CRP Evaluation study – 256 men and women without overt coronary disease, r: median values, s: Pravastatin Limitation of Atherosclerosis in the Coronary Arteries trial – 154 men and women with coronary heart disease, t: Pravastatin Limitation of Atherosclerosis in the Coronary Arteries trial – 130 men and women with coronary heart disease, u: Collaborative AtoRvastatin Diabetes Study – 69 type 2 diabetic men and women with modest dyslipidemia who had a previous myocardial infarction, v: New England Medical Center and University of Pennsylvania School of Medicine – 10 patients received placebo for four weeks then 120 mg torcetrapib daily for four weeks, 6 patients went on to receive 240 mg torcetrapib daily for an additional four weeks.

## Conclusion

Lowering LDL cholesterol is crucial to reducing cardiovascular morbidity and mortality [60], but LDL levels alone do not identify all patients at risk for coronary disease. Current evidence suggests that in addition to conventional plasma lipids, new measures of lipoprotein concentration, size, and composition may provide additional information for assessing cardiovascular risk. Therapeutic modulation of lipoprotein profiles, particularly LDL particle number and LDL size, appears to be associated with a significant reduction in CAD risk. Our data indicate that patients who participate in a lifestyle change program represent a unique population with a particularly atherogenic CAD risk factor profile who could benefit most from cardiovascular risk reduction. The Ornish Program was highly effective for men in altering traditional CAD risk factors and plasma lipoprotein profiles, which may reduce vascular reactivity in patients with established disease as well as those at high risk for cardiovascular events. Conversely, women showed little evidence for improvement in most physiological variables and no significant changes in lipoprotein profiles relative to controls. The beneficial effects of the program on plasma lipids and lipoproteins in men remained significant when controlling for the potential confounding effects of lipid-lowering medications. Additional studies are needed to determine if (1) improvements in lipoprotein profiles extend beyond one year and translate into improved clinical outcomes, (2) the apparent gender difference in lipoprotein response ultimately leads to improvement in long-term vascular health, and (3) gender tailored lifestyle prescriptions are required to confer optimal CAD risk benefit.

## Abbreviations used

ANOVA: analysis of variance; BMI: body mass index; BP: blood pressure; CAD: coronary artery disease; HDL: high-density lipoprotein; IDL: intermediate-density lipoprotein; LDL: low-density lipoprotein; NMR: nuclear magnetic resonance; sdLDL: small, dense LDL; TZD: thiazolidinedione; VLDL: very-low-density lipoprotein.

## Competing interests

The authors declare that they have no competing interests.

## Authors' contributions

DJD drafted the manuscript as a research physician; DMN established the program database, conducted statistical analysis, and assisted with data interpretation; AB directed the lifestyle change program, oversaw subject recruitment, and coordinated data collection; MJH recruited participants and was involved in data collection/entry; HLP obtained and processed blood samples from participants and drafted the manuscript; MNV critically reviewed the manuscript and contributed valuable expertise in clinical medicine; DLE conceived and designed the study, interpreted the data, and supervised manuscript preparation. All authors read and approved the final manuscript.
